# Predicting metabolic pathway membership with deep neural networks by integrating sequential and ontology information

**DOI:** 10.1186/s12864-021-07629-8

**Published:** 2021-09-27

**Authors:** Imam Cartealy, Li Liao

**Affiliations:** University of Delaware, Computer and Information Sciences, 101 Smith Hall, Newark, 19716 DE US

**Keywords:** Metabolic pathway prediction, Gene ontology, Neural network

## Abstract

**Background:**

Inference of protein’s membership in metabolic pathways has become an important task in functional annotation of protein. The membership information can provide valuable context to the basic functional annotation and also aid reconstruction of incomplete pathways. Previous works have shown success of inference by using various similarity measures of gene ontology.

**Results:**

In this work, we set out to explore integrating ontology and sequential information to further improve the accuracy. Specifically, we developed a neural network model with an architecture tailored to facilitate the integration of features from different sources. Furthermore, we built models that are able to perform predictions from pathway-centric or protein-centric perspectives. We tested the classifiers using 5-fold cross validation for all metabolic pathways reported in KEGG database.

**Conclusions:**

The testing results demonstrate that by integrating ontology and sequential information with a tailored architecture our deep neural network method outperforms the existing methods significantly in the pathway-centric mode, and in the protein-centric mode, our method either outperforms or performs comparably with a suite of existing GO term based semantic similarity methods.

## Background

Metabolic pathways are series of biochemical reactions occurring within the cell which involve catalytic reactions of protein enzymes converting substrate compounds into product compounds. Because each reaction in the pathway requires a protein enzyme as catalysis in order to happen, from an enzyme centric perspective, a metabolic pathway can be represented as a list of these proteins. Identification of organism’s metabolism usually involves laborious experimental techniques mainly in characterization of protein enzymes in metabolic pathways. It requires advanced technologies, expensive equipments, and highly skilled manpower to perform the experiments. To shorten the steps required in the characterization process, computational methods are often deployed for modeling the pathway and inferring specific tasks. The prediction step might provide a higher level of network organization that facilitate human comprehension of the system and aid in identifying the missing information such as missing proteins or reactions in the network. One example of such prediction tasks is pathway membership inference, which is to determine whether a protein is a member in the enzyme list of a given pathway. This is an important annotation task that can not only provide context to the basic function annotation of proteins but also more importantly aid reconstruction of incomplete metabolic pathways, which can subsequently help better understand metabolism and physiology of cells and provide complementary perspective to study evolutionary [1].

However, traditional sequence similarity-based homology approaches to characterizing proteins for their enzymatic properties run into difficulties when sequence identity is lower than 60% [2]. Facing this challenge, various efforts have been made to go beyond individual proteins and their homologs to leverage the large amount of annotations for proteins in their functional context, such as from curated reference dataset or features extracted from proteins. The example of curated reference dataset is Gene Ontology (GO), which provide a hierarchy of controlled terms defining protein functions with varied levels of specificity for different cellular functions/processes [3, 4]. The semantic similarity between two proteins can be used to replace the sequence-based similarity method.

Various similarity measures have been developed to quantify the semantic similarity of GO terms and applied it in quantitative comparison of functional similarity of gene products, although most of these methods are not developed for metabolic pathway membership inference [5–10]. Essentially, those measures mainly involve two steps of calculation : 1) calculation of GO term similarity, and 2) calculation of protein similarity, based on GO term similarity. In the first step, the semantic similarity between two GO terms is calculated to incorporate the GO hierarchy, via information contains in the GO tree such as node, edge or combination of the two. In the second step, protein similarities are aggregated from their terms’ similarities. To infer the protein’s membership in the pathway, the similarity between the proteins are then used [7, 11]. More recently, in [5], a hybrid approach to take into account of both information content of individual GO terms and the whole GO hierarchy with a simple Cosine similarity is shown to be advantageous in both prediction accuracy and running time as compared with other semantic similarity-based methods.

In general, however, the prediction task of proteins’ annotation, including the prediction of protein’s metabolic pathway annotation, may come from two perspectives. One perspective is the pathway centric perspective and the other is protein centric perspective. In the pathway centric perspective, the relevant question is: given a pathway, predict the proteins participate in the pathway, thus this perspective leads to prediction problem of association of pathway and its enzymatic reaction. On the other hand, the protein centric problem asks a different question: given a protein and its annotation, predict enzymatic reaction that they catalyzed. This question can be translated into prediction of set of metabolic pathways of which a given protein is likely to be a member. While the protein centric perspective is more natural in protein annotation, it turns out more computationally challenging as it is multi-class classification problem, as compared to the binary classification problem for pathway centric membership prediction.

In this work, we set out to develop new computational approach based on neural networks for predicting pathway membership from both directions: the protein centric and pathway centric problems. In doing so, we also explore integrating both ontology and sequential information to further improve the accuracy. Specifically, we develop a neural network model with an architecture tailored to facilitate the integration of features from different sources.

## Results and discussion

Table 1 shows the performance of our method for pathway membership prediction, in comparison to using a suite of different ontology-based gene similarity methods mentioned in the Methods. Because GO has three separate hierarchies: BP, CC, and MF, we thus evaluated the prediction performance for using each hierarchy. In addition, we also evaluated the performance of different featured used in this experiment separately.

**Table 1:** The ROC score of different methods in pathway membership prediction for all ontologies. NN is neural network model, NN 1/0 is neural network model that use binary representation of GO terms as features. The number of layers in neural network are three and the

Methods	BP	CC	MF
NN	0.953	0.849	0.895
NN 1/0	0.941	0.847	0.870
cosine	0.931	0.762	0.677
SVM	0.920	0.768^†^	0.843
RF	0.935^†^	0.837	0.887
KNN	0.830	0.780	0.806
Resnik	0.829	0.667	0.790
SimGIC	0.902	0.735	0.717

We developed a method to include the graph structure information of gene ontology and the information contain in ontology terms as feature representation of proteins. The inclusion of both graph structure and information content in our method can significantly improve performance of pathway prediction membership. When a simple approach of binary vector 1 or 0 to represent the presence of GO term for a given protein, the performances of prediction are lower than our method for all ontologies, for example when BP ontology is used the performances are.941 and.953 respectively (statistically significant, p <0.05)

In comparison across three ontologies, the best results are obtained when BP ontology terms are used as features to predict the membership of metabolic pathway for all methods. It is clear that the neural network model outperformed other methods. For example, when BP terms are used, the ROC score for neural network,,cosine, SVM, RF, and KNN are.953,.931,.920,.935, and.830 respectively. When cosine method is used as a baseliner method, our method’s performance is statistically significant higher (p <0.05), while other machine learning methods such as KNN and SVM are lower. However, it is interesting to note that the performance of methods that are designed specifically to use the ontology-based semantic similarity such as SimGIC, and Resnik, are mostly the worst performance in all ontologies, even below the baseline cosine method. The reason behind this may be explained by the fact that most of ontology-based semantic similarity methods are based on calculating the similarity distance between the proteins only, without the learning process such as SVM classifier.

The good performances of prediction methods when using GO terms ontology are expected since the GO terms are curated data. The BP terms are especially information rich of protein function dataset. Other ontology terms, i.e. MF and CC, are not as rich as the BP in terms of function information, thus the performance of methods in predicting protein membership of pathway when using these ontologies are below the BP ontology. This pattern is consistent with our intuition that metabolic pathways are better characterized as biological processes (BP). Realizing this, we tested the performances of neural network method and base classifier when using non function based curated data, such as k-mer which transform the sequence information into frequency of k-mer amino acids, as input features to the models. Compared to the performances when GO terms are used as features, the sequence-based features are less effective in pathway membership prediction task (Table 2). The top model performance when using this feature are.786 for neural networks model.

**Table 2:** ROC score of different methods when k-mer is used as input features

Methods	k-mer
NN	0.786
cosine	0.598
SVM	0.715
RF	0.774
KNN	0.687

We also tested the effect of multi modal features as input to our neural network model. We tested two different possibilities of combining the multi modal features in our NN model, by concatenating the features at early stage and at later stage. Addition of information to the method can improve the prediction performance of NN model (Table 3), although in other models it can lower the prediction performance. For example, compare to single modal of GO term in NN architecture, the use of multi-modal data can increase the performance from.952 to.957 (p=0.17), from.849 to.880 (p <0.05), and from.895 to.907 (p <0.05) when BP, CC, and MF ontologies are used. However, in cosine method, the use of multi-modal data of GO terms and k-mer frequency can deteriorate the prediction performance. We believe this attributed to the learning power of the neural network, in which individual neurons can adjust their weights adapting to different type of features, whereas the cosine method treats all features equally.

**Table 3:** The ROC score of methods for multi-modal data. NN is neural network model, NN 1/0 is neural network model that use binary representation of GO terms as features. (concat) is approach where GO terms and k-mer is concatenated as single vector to represent each protein, (multi-input) approach where GO terms and k-mer are used as two input to the model. The number of layers in neural network are three and the dimension of neurons in each layer are 128,64, and 1

Methods	BP+k-mer	CC+k-mer	MF+k-mer
NN (concat)	0.954	0.874	0.907
NN (multi-input)	0.957	0.880	0.907
NN 1/0 (concat)	0.943	0.883	0.894
NN 1/0 (multi-input)	0.940	0.882	0.888
cosine (concat)	0.920	0.768	0.687
SVM (concat)	0.933	0.814	0.863
RF (concat)	0.923^†^	0.840	0.844
KNN (concat)	0.829	0.784	0.790

^†^not significantly different than cosine method in each ontology

When we considered the metabolic membership prediction task as a pathway centric problem, we needed to build many models, one for each pathway. Thus, for a given protein to be classified, we need to run it for every model and obtained the predicted output. The protein centric prediction task, on the other hand, will predict multiple classes at once thus can be built from one model. Table 4 shows the performance of neural network method in comparison to other methods by using either single modal or multi modal features.

**Table 4:** The performance comparison of models in protein centric task. The table reports the true positive (TP), false negative (FN), false positive (FP), number of proteins that have at least 1 prediction label (NP), the precision, recall, F measure, and Matthews correlation coefficient (MCC) for different features used in the models. The features used are k-mer, GO terms (BP, CC, and MF), and when both k-mer and GO terms are combined. The number of layers in neural network are three where the dimension of the first two layers are 128 and 64, and the last layer dimension is equal to the number of metabolic pathways

Features	Method	TP	FN	FP	NP	Precision	Recall	F1 measure	MCC
k-mer	NN	1312	4183	3481	920	0.216	0.178	0.195	0.246
	SVM	206	5289	41	149	0.823	0.074	0.136	0.176
	RF	201	5294	0	71	1.000	0.052	0.099	0.190
	KNN	792	4703	457	332	0.624	0.132	0.217	0.300
BP	NN	2773	2722	2256	984	0.646	0.513	0.572	0.521
	SVM	2283	3212	711	888	0.796	0.449	0.574	0.560
	RF	1709	3786	170	623	0.906	0.375	0.531	0.529
	KNN	1391	4104	424	787	0.755	0.374	0.500	0.438
BP+k-mer	NN	2760	2735	1830	973	0.648	0.500	0.565	0.544
	SVM	2301	3194	709	883	0.804	0.448	0.575	0.563
	RF	1198	4297	42	371	0.970	0.227	0.368	0.456
	KNN	1430	4065	394	773	0.764	0.375	0.503	0.449
CC	NN	1945	3550	3616	926	0.422	0.355	0.386	0.343
	SVM	1117	4378	283	534	0.768	0.255	0.383	0.400
	RF	1009	4486	267	445	0.736	0.215	0.333	0.379
	KNN	966	4529	436	525	0.627	0.204	0.308	0.346
CC+k-mer	NN	2178	3317	2523	827	0.493	0.348	0.408	0.421
	SVM	1213	4282	302	551	0.784	0.270	0.401	0.418
	RF	659	4836	25	212	0.977	0.132	0.232	0.338
	KNN	1026	4469	450	535	0.675	0.224	0.336	0.358
MF	NN	2429	3066	2950	844	0.545	0.400	0.462	0.439
	SVM	1703	3792	423	646	0.785	0.306	0.441	0.496
	RF	1580	3915	454	604	0.786	0.316	0.451	0.470
	KNN	1313	4182	576	635	0.642	0.262	0.372	0.405
MF+k-mer	NN	2520	2975	2900	868	0.580	0.399	0.472	0.454
	SVM	1771	3724	449	665	0.783	0.326	0.460	0.504
	RF	985	4510	18	275	0.968	0.157	0.270	0.417
	KNN	1427	4068	533	612	0.697	0.272	0.391	0.432

Similar to pathway-centric prediction task, the performances of the protein-centric methods are best when BP ontology is used as feature. The F measure of NN for example, are.572,.386, and.462 when BP, CC and MF ontology are used respectively. When NN model being compared to other classifiers, it outperforms most of the classifiers, especially when using the MF and CC dataset, while when using BP dataset, it is second under SVM classifier. However, it is important to note that of all classifiers, neural network produced the highest number of proteins that have at least one predicted label in all ontologies and highest number of true positive, which suggest that the neural network being more sensitive (thus higher recall) in detecting the metabolic pathway to the proteins, while other classifiers are more being specific (hence higher precision). Consequently, NN produces highest number of false positive and lowest number of false negative of all methods, while SVM produces lower false positive and higher false negative than NN. Overall, however, as measured by the F1 score that takes into account both recall and precision, NN either outperforms other methods (CC, CC+k-mer, MP, MP+k-mer)or performs comparably with other methods (BP, BP+k-mer). It is worth noting that, the protein-centric membership prediction is a multi-class classification whereas the pathway-centric membership prediction is a binary classification, which means that the former one is much more challenging, as reflected in the prediction performance. Therefore, while performance for protein-centric membership prediction may seem low, it should be assessed in the context of multi-class (320 classes to be exact) classification with a 1/320 = 0.3% accuracy from a random classifier.

## Conclusion

In this work, we developed a neural network-based method for pathway membership inference using both gene ontology (GO) similarity and sequential features between a query protein and proteins that are known to the members of a given pathway. By replacing binary vector of the GO term annotation for a gene with the information content of individual GO terms and incorporating GO hierarchy with ancestor nodes that are directly present in gene annotation, we can create information rich vector representation for a gene. We built multilayer forward feeding neural networks that are able to integrate the GO term features and sequential features. We demonstrated that our NN based method outperformed other classifiers including SVM and random forest and the methods that are specifically designed to use the GO term features alone. Moreover, the NN based method is also able to answer question from both the pathway centric and protein centric perspectives, which makes the method more versatile in scaled up application for protein annotation.

## Methods

### Dataset

We used the gene ontology and gene annotation from GeneOntology (GO, http://geneontology.org), version 2019-07-01. The GO’s ontology consists of three ontologies, i.e. biological process (BP), cellular components (CC) and molecular functions (MF). This version of GO contains 31043 BP, 11973 MF, and 4397 CC terms. The annotation provides association between proteins and their corresponding GO terms either manually reviewed by curator or automatically generated by prediction tools. Out all of available evidence codes, only IEA (Inferred from Electronic Annotation) has not assigned manually by a curator. Therefore, it is necessary to exclude the IEA evidence code to prevent cyclic prediction: predict the protein annotations by using predicted data. In this experiment, we exclude annotations encoded by IEA.

**Fig. 1:**
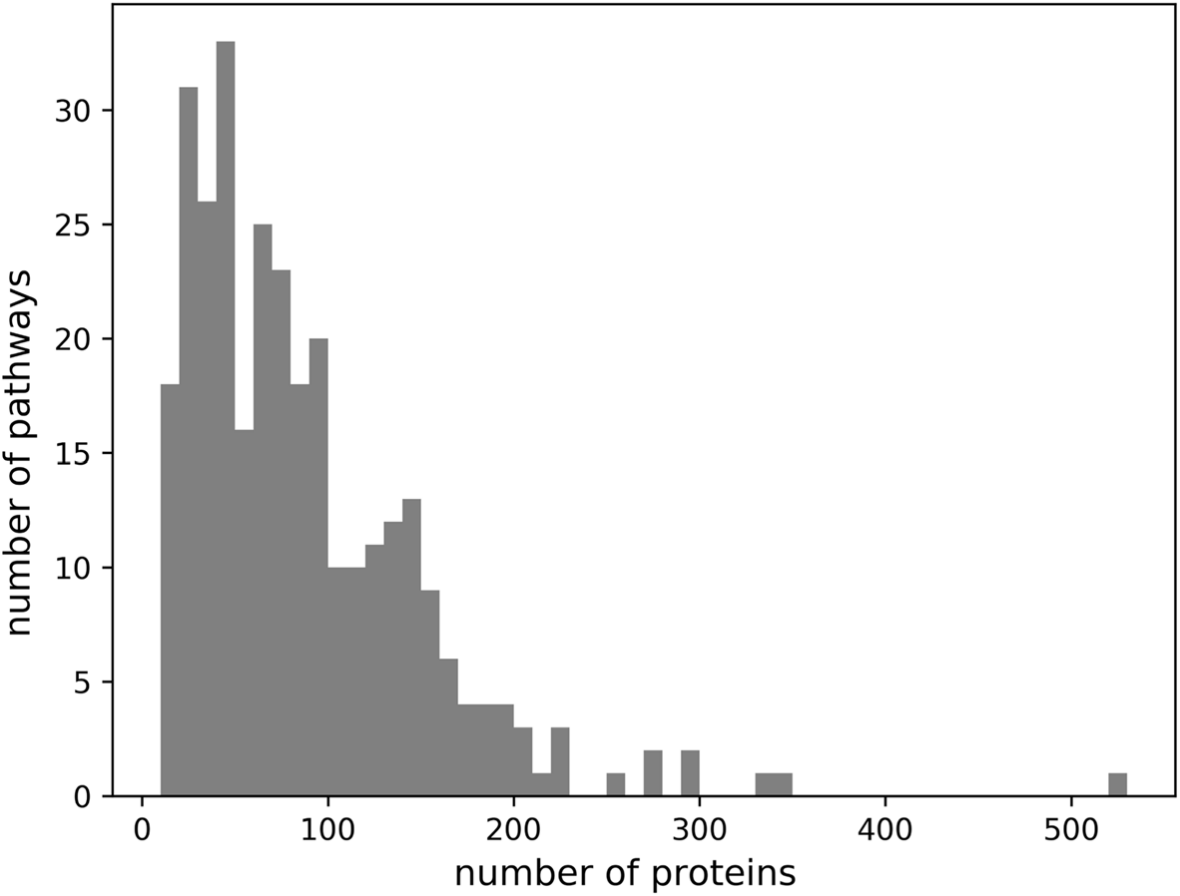
Distribution of pathways and the number of their proteins used in this experiment

We downloaded human KEGG pathway data set from Kyoto Encyclopedia of Genes and Genomes database [12], http://rest.kegg.jp. The database consists of 320 human pathways. We excluded pathways that consists less than 10 proteins to ensure adequate training and testing in the cross-validation scheme and mapped the NCBI gene id to its corresponding Uniprot identifier. As a result, we obtained 308 pathways and the number of proteins in the pathways range from 10 to 521 proteins with most of the pathways having proteins less than 100 proteins (Fig. 1).

### Data representation

**Fig. 2:**
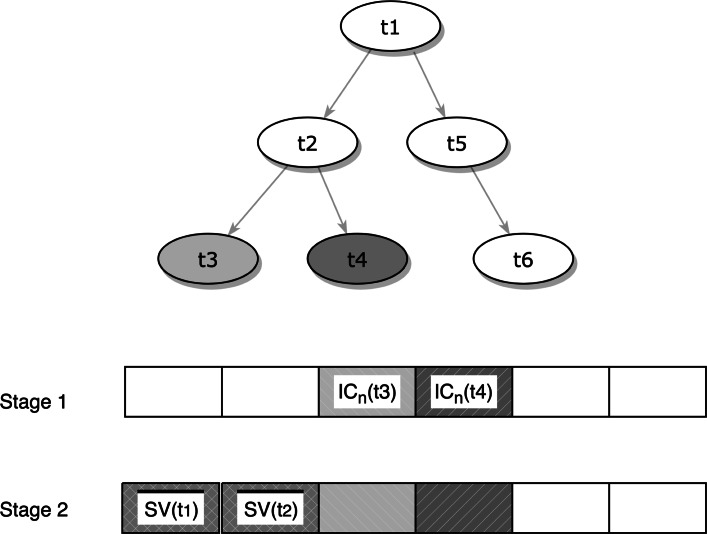
Generation of vector representation from GO dataset. In this example, the protein is annotated with t3 and t4. To generate protein’s feature vector, the normalized IC of t3 and t4 is used in the first stage. On second stage, the semantic value (SV) of all term ancestors of t3 and t4 are calculated. Since t3 and t4 share common ancestor, t1 and t2, the semantic value for t1 and t2 are average semantic value ($\overline {SV} $). See Material and Methods for detail description

We used multimodal data as input to our model, including the GO terms and k-mer information from protein sequences. While a simplistic approach to represent GO terms is a binary vector with 1 or 0 representing the presence or absence of GO terms in annotation of given gene, our method adopts a scheme from [5], which considers both of the structure of the GO graph and the information content of the GO terms in building the vector of the gene and their corresponding annotations (Fig. 2).

Specifically, before we build the gene vector, we first calculated the semantic value (SV) for each GO term in the annotation of a given protein. We used a normalized information content of term *t*_*i*_ by dividing the information content of term *t*_*i*_ with the maximum IC in whole set of GO terms *T* as follows:

1 $$ IC_{n}(t_{i}) = \frac{IC(t_{i})}{ \max\limits_{t_{j} \in T} IC(t_{j})}  $$

Then we expanded the annotation of a given protein by including all of the ancestor terms: for each annotation term *t*_*i*_ in a given protein, we assigned the weighted semantic value for all ancestor terms of term *t*_*i*_, defined as follows:

2 $$ SV(t_{{pi}}) = w^{d_{p}}IC_{n}(t_{{pi}}),  $$

where*w* is the weight, in this case we use a fix constant of 0.5, *t*_*pi*_ is all ancestor terms of term *t*_*i*_ and *d*_*pi*_ is the path length of term *t*_*i*_ to its ancestor *t*_*pi*_. The path length is defined as the difference of the maximum depth between the two terms in the GO tree.

When there are multiple GO terms in the annotation of a given protein, it is possible that these GO terms may have ancestor terms in common. Therefore, during expansion of the annotation vector for a given protein, a common ancestor term will have multiple semantic values, each for annotation term in the original annotation, as the common ancestor term may receive a semantic value from all of its descendants. Hence, we calculated the average of these values ($\overline {SV(t_{p})} $) as the new semantic values for a common ancestor term *t*_*p*_. Note that, in GO hierarchy, there are other relationships such as “NOT” and “contribute to”, between two GO terms; in this study, however, we only include “is_a” relationship for calculating the semantic value, following the same practice as in other method such as [7], which we compare with.

After this procedure, a gene is represented as a vector of n-dimension, where n = |*T*|, each dimension corresponding to one GO term in the gene ontology hierarchy, with a semantic value being either a) the normalized information content if the GO term is present in the gene annotation, or b) a value assigned as above for a GO term whose descendant(s) is present, or c) a value of zero if a GO term is not of either of the two former cases.

In addition to gene annotation data as input to our model, we also used sequence-based features, such k-mer. The k-mer feature represents the sequence information as the frequency of k-mer, in this case we used *k*=2.

### Neural network architecture

Artificial neural network is inspired by biological process [13]. It consists of layers of neurons that are fully connected between layers, but no connection between neurons in the same layer. Each neuron performs linear transformation operation of weighted information summation coming from all neurons in previous layer adjusted by some biases followed by nonlinear activation function *f*, as define by following equation:

3 $$ x = f \left(\sum{w_{i} x_{i}} +b \right)  $$

While there are many activation options available for neural network. The two most used activation functions are ReLU and Sigmoid. The ReLu set the lower bound output of neuron to 0 the output of neuron to be minimum of 0, while sigmoid squashing the output of neuron and bounded to be between 0 and 1. In this experiment, we used the ReLU activation in the hidden layer, while Sigmoid is used in the output layer. The formal definition of ReLU (4) and Sigmoid (5) are:

4 $$  y = max (0,x)  $$

5 $$  y = \frac{1}{1+ e^{(-x)}}  $$

**Fig. 3:**
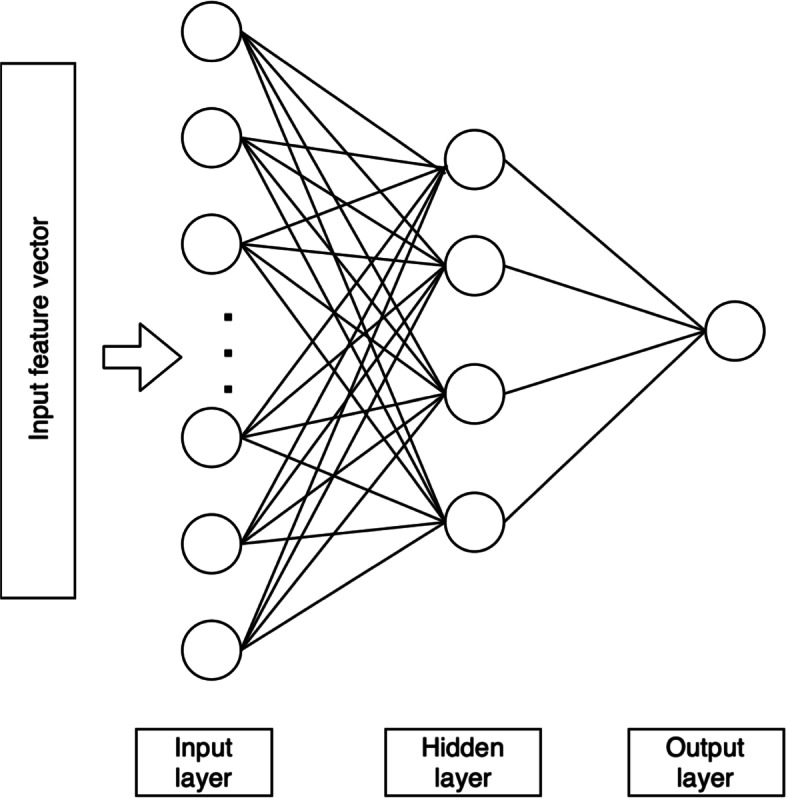
Neural network architecture for single vector input

**Fig. 4:**
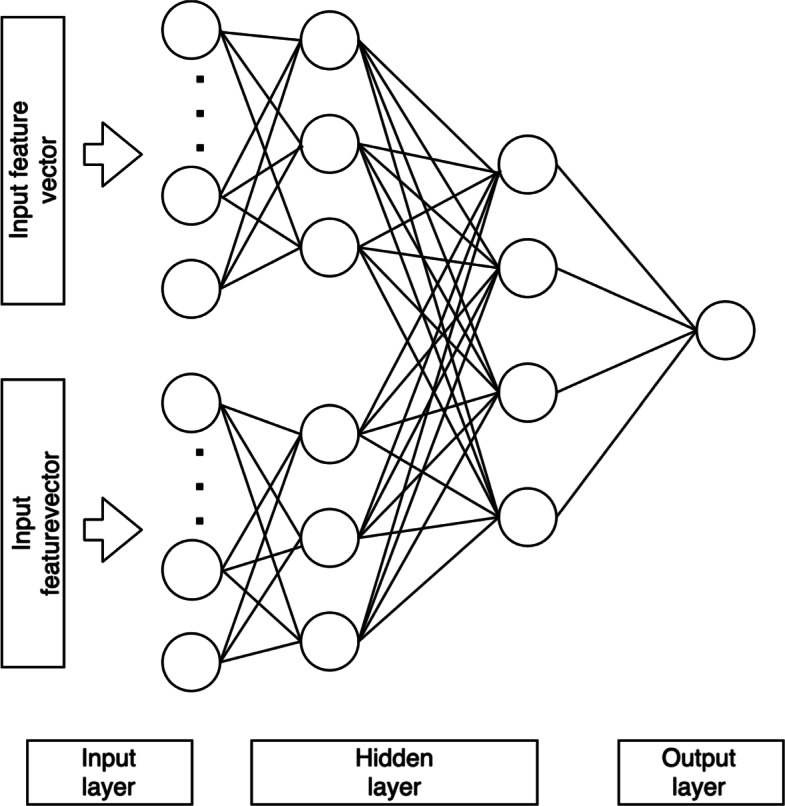
Neural network architecture for multi input vector

We implemented a multi-layer feed forward deep neural network in our model. We stacked three fully connected layers where the first layer is the input layer, the second layer is hidden layer, and the last layer is output layer. The input of the network is the n-dimensional vector of protein’s features (Fig. 4). We used multi-modal features, i.e. GO terms and k-mer, and we either used a single modal or multi-modal features. For a single modal feature, we adopt architecture in Fig. 3. For multi-modal features, we combined the features’ vectors at early stage or at later stage. At early stage, we concatenate multiple vectors into one vector as input to the model, thus the architecture similar to single input vector (Fig. 3). On the other hand, the concatenation at later stage happens inside the model where multi input model accept multiple input of vectors, then the model combine it in hidden layer while processing the inputs (NN multi input, Fig. 4). Note that convolution neural networks were attempted and did not get good performance, which we believe may be attributed to lack of convoluted patterns/features in protein sequences, unlike 2d images. Depending upon the classification task, the dimension of output layer is either 1 or *n*, where *n* is the number of classes to be predicted (*n*=308). In binary classification, the dimension of output layer is 1, while in multi-label classification the dimension of output layer is *n*. For binary classification task, we built one model for each class, while for multi-label classification task, we built one model. We performed optimization by comparing different number of neurons in each layer (data not shown).

We implemented the Keras library to build our model. We chose to minimize the binary cross entropy function loss using the Adam optimizer with learning rate 0.001 for binary classification task. For multi label classification task, we chose to minimize the F1 function loss. To prevent overfitting in our model, we implemented the dropout (0.5) regularization. Note that unless explicitly mentioned otherwise, the default values of the hyperparameters are used in this study, and it is conceivable that better performance than reported in Tables 1, 2, and 3 can be achieved should these hyperparameters be optimally tuned.

### Training

In our experiment, we trained individual model separately for each pathway in the binary classification task. We performed 5-fold cross validation for each pathway. For each pathway, positive dataset consists of proteins that belong to the pathway while negative dataset is generated by selecting equal number of random proteins that do not belong to the pathway or interacting with proteins in the pathway. We followed this procedure since proteins in the pathway tend to interact each other, and by using this approach we ensured that there are no proteins in the negative dataset that are interacting with proteins in the positive dataset. We used BioGrid dataset to determine the interacting protein. We also excluded proteins that have no GO terms information in the pathways.

For multi-label classification task, we followed different approach. Since in both multi class or multi label classification task a positive sample can be a negative sample for other classes, we did not generate negative dataset. We simply consider negative dataset of a given pathways are proteins in other pathways. We also did not perform 5-fold cross validation, instead we randomly held 5 proteins from each pathway as testing dataset and the rest as training dataset.

### Baseline classifiers

We used several GO based semantic similarity measures and baseline classifier as comparison to our method. We used the most commonly used semantic similarity measures, Resnik [10] and simGIC [9]. These measures mainly use the information content (IC) of each node to quantify the GO terms in the GO graph. The IC is described as:

6 $$ IC(t) = -log[p(t)]  $$

where *p*(*t*) is term frequency of *t* in a given annotation corpus, such as Gene Ontology Annotation (GOA). These measures use same principal in calculating similarity between two proteins, which is based on the similarities of their corresponding terms. For protein similarities of Resnik’s measures, we followed method from [14]. In addition to these methods, we also calculated the similarity of two proteins *q* and *p* based on their dot product of their corresponding semantic value vectors *SV*_*q*_ and *SV*_*q*_ as

7 $$ s(q, p) = \sum_{t \in T} SV_{q}(t) \cdot SV_{p}(t)   $$

where *t* is the term of GO terms *T*. To determine whether query protein *q* belong to the model, we used the average similarity score between the query protein and set of proteins *P* of incomplete pathways as

8 $$ S(q,P) = \sum_{p \in P} S(q, p) / |P|  $$

where *s*(*q*,*p*) is the similarity score between query protein q and a member protein p as calculated by Eq. 7 and |*P*| is the number of known proteins for the incomplete pathway P.

In addition to GO based semantic similarity methods, we also use some of mostly used base classifiers in machine learning: SVM, RF, and KNN. We implemented the Scikit library of SVM, RF, and KNN by using all default parameters. We used parameters as follows: rbf kernel and *C*=1*e*10 in SVM, number of forest is 100 in RF, and number of neighbor is 5 in KNN. We implemented Scikit SVM, RF, and KNN libraries.

### Predictive performance evaluation

We adopted two different performance measures, each for pathway centric and protein centric prediction task respectively. For pathway centric task, we considered the task as binary classification problem and used receiving operating characteristic (ROC) curve analysis to evaluate the performance. The ROC curve of perfect classifier has the area under the ROC curve (AUC) of 1. The perfect curve rises steeply from bottom left to top left and move toward top right. We calculated ROC curve for each pathway and average across all pathways. ROC curve measures the performance of classifier at various threshold setting and represents the tradeoff between true positive rate (TPR) and false positive rate (FPR). The TPR and FPR for each pathway *c* are defined as:

9 $$ FPR_{c} = \frac{FP_{c}}{(FP_{c}+TN_{c})}  $$

10 $$ TPR_{c} = \frac{TP_{c}}{(TP_{c}+FN_{c})}  $$

where *FP*_*c*_,*TN*_*c*_,*TP*_*c*_, and *FN*_*c*_ are the number of false positive, true negative, true positive and false positive respectively in pathway *c*. We then calculated the AUC of ROC from the above *FPR* and *TPR* and average the ROC score over all pathways.

For protein centric task, we considered the task as multi-label classification since one protein can have multiple label, and used the F1 score and Matthews Correlation Coefficient (MCC) to evaluate performance. The precision and recall are defined as

11 $$ p = \frac{TP}{TP+ FP}  $$

12 $$ r = \frac{TP}{TP+ FN}  $$

where *TP*, *FP*, and *FN* are the number of true positive, false positive, and false negative respectively. The F measure is harmonic mean of precision and recall. The value range between 0 and 1. The perfect score of 1 means that both of the precision and recall reach their maximum score of 1. However, when the precision reach maximum, it increases the TN, thus reducing the recall. On the other hand, when the recall reaches maximum score, it increases the FP, thus reducing the precision. Thus, F measure hardly reach maximum score 1. The F1 measure is defined as

13 $$ F1 = \frac{2 \times p \times r}{p+r}  $$

while MCC is defined as:

14 $$ \begin{aligned} MCC = \frac{(TP \times TN) {-}(FP \times FN)} {\sqrt{ (TP+FP)\times(TP+FN)}{\times(TN+FP)\times(TN+FN)}} \end{aligned}  $$

## Data Availability

The data and the code used in this study will be made available to the readers for free upon request. The complete and updated data of GO annotations and KEGG pathways can be accessed from http://geneontology.org and http://rest.kegg.jp respectively. Declarations

## Abbreviations

GO: Gene ontology
KEGG: Kyoto encyclopedia of genes and genomes
KNN: K-Nearest neighbors
NN: Neural networks
RF: Random forest
ROC: Receiver operating characteristic
SVM: Support vector machines
